# Distinct Phenotype, Longitudinal Changes of Numbers and Cell-Associated Virus in Blood Dendritic Cells in SIV-Infected CD8-Lymphocyte Depleted Macaques

**DOI:** 10.1371/journal.pone.0119764

**Published:** 2015-04-27

**Authors:** Caroline Soulas, Patrick J. Autissier, Tricia H. Burdo, Michael Piatak, Jeffrey D. Lifson, Kenneth C. Williams

**Affiliations:** 1 Department of Biology, Boston College, Chestnut Hill, Massachusetts, United States of America; 2 AIDS and Cancer Virus Program, Leidos Biomedical Research, Inc., Frederick National Laboratory, Frederick, MD 21702, United States of America; Emory University School of Medicine, UNITED STATES

## Abstract

Loss of circulating CD123+ plasmacytoid dendritic cells (pDCs) during HIV infection is well established. However, changes of myeloid DCs (mDCs) are ambiguous since they are studied as a homogeneous CD11c+ population despite phenotypic and functional heterogeneity. Heterogeneity of CD11c+ mDCs in primates is poorly described in HIV and SIV infection. Using multiparametric flow cytometry, we monitored longitudinally cell number and cell-associated virus of CD123+ pDCs and non-overlapping subsets of CD1c+ and CD16+ mDCs in SIV-infected CD8-depleted rhesus macaques. The numbers of all three DC subsets were significantly decreased by 8 days post-infection. Whereas CD123+ pDCs were persistently depleted, numbers of CD1c+ and CD16+ mDCs rebounded. Numbers of CD1c+ mDCs significantly increased by 3 weeks post-infection while numbers of CD16+ mDCs remained closer to pre-infection levels. We found similar changes in the numbers of all three DC subsets in CD8 depleted animals as we found in animals that were SIV infected animals that were not CD8 lymphocyte depleted. CD16+ mDCs and CD123+ pDCs but not CD1c+ mDCs were significantly decreased terminally with AIDS. All DC subsets harbored SIV RNA as early as 8 days and then throughout infection. However, SIV DNA was only detected in CD123+ pDCs and only at 40 days post-infection consistent with SIV RNA, at least in mDCs, being surface-bound. Altogether our data demonstrate that SIV infection differently affects CD1c+ and CD16+ mDCs where CD16+ but not CD1c+ mDCs are depleted and might be differentially regulated in terminal AIDS. Finally, our data underline the importance of studying CD1c+ and CD16+ mDCs as discrete populations, and not as total CD11c+ mDCs.

## Introduction

Dendritic cells (DCs) are professional antigen presenting cells with the unique ability to present antigens to naïve T cells, inducing adaptive immune responses and controlling tolerance and immune activation [1]. Thus it is likely that DCs play a role in the control of human immunodeficiency virus (HIV) infection and development of acquired immune deficiency syndrome (AIDS). Peripheral blood DCs in humans and monkeys are usually defined, using 4–5 color flow cytometry, as two major populations: lineage (Lin)- HLA-DR+ CD11c+ CD123- myeloid DCs (mDCs) and Lin- HLA-DR+ CD11c- CD123+ plasmacytoid DCs (pDCs). It is well established that absolute numbers of blood CD123+ pDCs decrease during HIV and SIV infection [2–4] but the effects of HIV/SIV infection on mDC numbers are less well defined. Some reports show decreased numbers of mDCs during chronic HIV and SIV infection [4–8] while others have demonstrated increased numbers of mDCs in SIV-infected rhesus macaques [9]. The correlation between absolute numbers of DCs and plasma virus or CD4+ T lymphocyte counts has been studied but the results are inconsistent [10–12]. Whether circulating or resident tissue DCs are actively HIV and SIV infected is also a matter of debate [13–16].

Monitoring DC numbers and infection is challenging due to cell heterogeneity, low cell numbers, and technical differences in immune phenotype and detection. In addition, conflicting data on modulation of DC numbers in AIDS exist due to discrepancies in the specimens studied (acute vs. asymptomatic vs. chonic stages of HIV infection, and whether or not patients are on ART). For these reasons, non-human primate models of AIDS represent a more comprehensive way to study kinetics of DC subsets and viral infection.

Non-overlapping subsets of mDCs that are CD1c+ mDCs and CD16+ mDCs have been identified in non-infected humans and rhesus macaques. These subsets are distinguished phenotypically and functionally [17–20]. CD1c+ mDCs that secrete high levels of IL-8 might be involved in monocyte chemotaxis, while CD16+ mDCs secreting high levels of TNF-alpha might be stronger pro-inflammatory cells [20].

Because these mDC subsets may have different immune roles in infection and they have not been studied as discrete populations in AIDS, we studied them throughout infection in SIV-infected CD8+ lymphocyte depleted rhesus macaques as this model allows a significant and rapid increases of viremia, rapid progression to AIDS and reduced survival of over 95% of SIV-infected CD8 depleted animals in a short time period (3–4 months pi) [21]. Using this model, we have published immunologic findings including evelvated sCD163 in plasma, expanssion of CD14+CD16+ monocytes in blood, accumulation of CD163+ macrophages in cardiac and CNS tissues, that have subsequenstly been demonstrated in chronically HIV infected individuals on durable combination anti-retroviral therapy (cART) [22–25]. Using a single 11-color flow cytometry panel, we studied changes in CD1c+ mDCs, CD16+ mDCs, and CD123+ pDCs in primary infection and until the development of AIDS. In addition, we analyzed FACS-purified subsets for SIV-RNA and-DNA at early and late time points. We found a decrease in all three subsets in the first week of infection, and CD123+ pDCs remained depleted while the CD1c and CD16+ mDC numbers returned to normal levels within three weeks. With development of AIDS, numbers of CD123+ pDCs and CD16+ mDCs were significantly lower than their pre-infection levels, which was in contrast to the CD1c+ mDCs. These data suggest a differential modulation of CD1c+ mDCs versus CD16+ mDCs with disease. We detected genomic SIV gag-RNA in all populations as early as 8 dpi, but SIV gag-DNA was only detected in CD123+ pDCs at 40 dpi, suggesting that mDCs harbor SIV-RNA on the surface or in endocytic compartments while pDCs are potentially productive viral reservoirs.

## Results

### Blood CD123+ plasmacytoid DCs, and CD1c+ and CD16+ myeloid DCs in rhesus macaques

**Fig 1:**
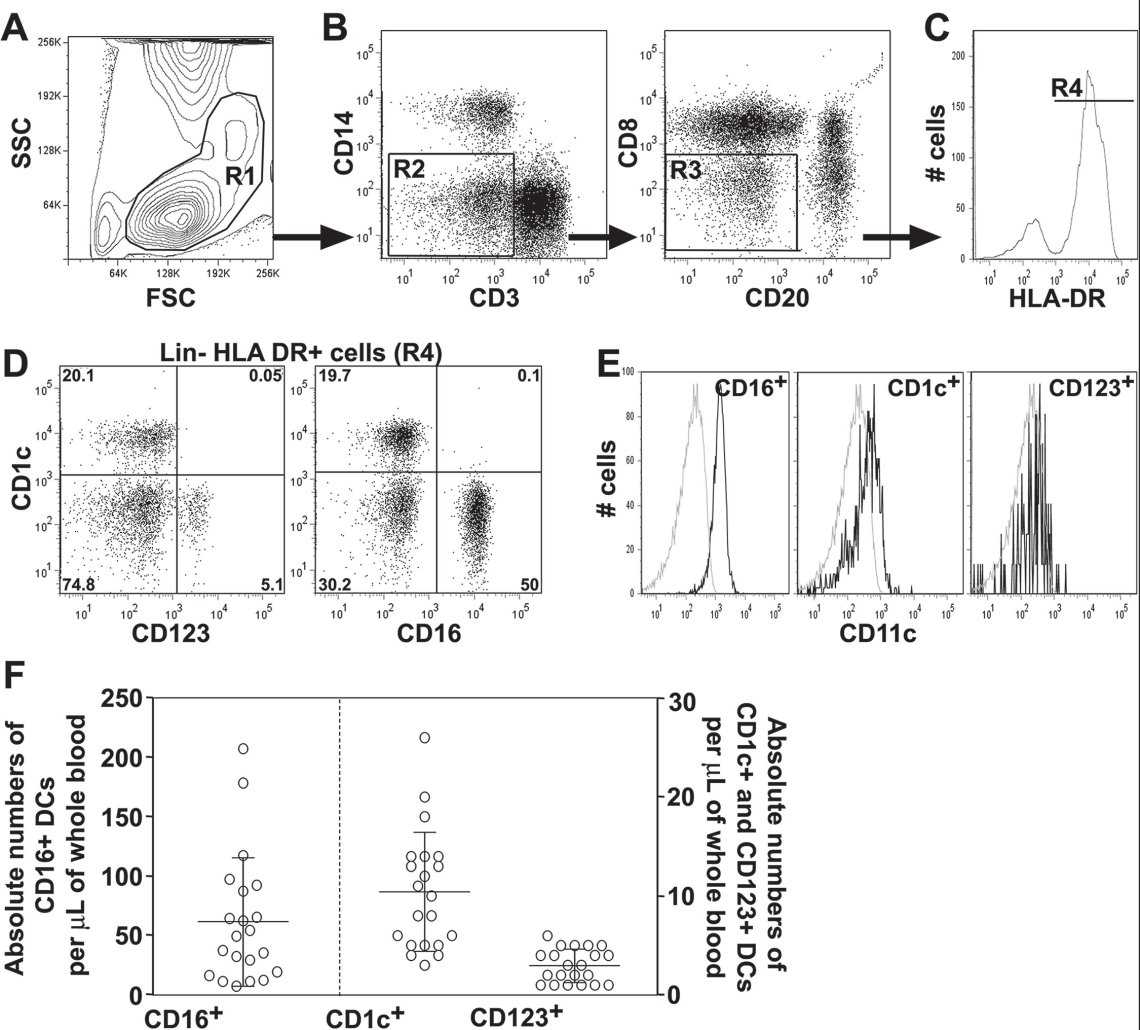
CD1c+ and CD16+ mDCs, and CD123+ pDCs in whole blood from rhesus macaques. Upper row: DCs are initially identified based on forward and side scatter (FSC and SSC) profiles of whole blood (**A**; R1 gate). CD3+ T lymphocytes and CD14+ monocytes are excluded (**B**; R2 gate) as well as CD20+ B lymphocytes and CD8+ NK cells (**B**; R3 gate) resulting in the selection of a Lin- population. From this, HLA-DR+ cells are selected (**C**; R4 gate). **D**. Within Lin- HLA-DR+ cells, non-overlapping CD1c+ and CD16+ mDCs and CD123+ pDCs are identified. Percentages of positive cells from Lin- HLA-DR+ cells are indicated. **E.** CD11c is expressed on CD16+ mDCs and at lower levels on CD1c+ mDCs. CD123+ pDCs were negative for CD11c. Grey histograms represent a negative control as CD11c expression by CD3+ T lymphocytes; black histograms show CD11c expression on CD1c+, CD16+ and CD123+ DC. Data are from animal 244–96 analyzed before infection representative of 21 normal rhesus macaques. **F**. Absolute numbers of DCs per microliter of whole blood determined in normal rhesus macaques. CD16+ mDCs represents the major population of DCs in whole blood of healthy rhesus macaques (left graph and left y-axis). CD1c+ mDCs and CD123+ pDCs are present in lower proportions (right graphs and right y-axis). Data are mean±SD from 21 uninfected animals.

Based on defined subsets of human blood DCs [17,18], we developed a single multicolor-flow cytometry panel that identifies three non overlapping circulating DC subsets in rhesus macaques [19]. DCs were first selected using forward and side scatter properties as cells between lymphocytes and monocytes (Fig 1A; gate R1). Lineage negative (Lin-) cells were selected by excluding CD3+ T lymphocytes and CD14+ monocytes (Fig 1B; gate R2), and CD20+ B lymphocytes (Fig 1B; gate R3). CD16+ NK cells were excluded by gating on CD8- cells (Fig 1B; gate R3) and HLA-DR+ cells (Fig 1C; gate R4) as previously described [19]. From the Lin- HLA-DR+ cells, three non-overlapping DC subsets were identified: Lin- HLA-DR+CD1c-CD16-CD123+ (CD123+) pDCs and two mDC subsets: Lin- HLA-DR+CD1c+CD16-CD123- (CD1c+) and Lin- HLA-DR+CD1c-CD16+CD123- (CD16+) mDCs (Fig 1D). Similar to humans, rhesus macaque CD123+ pDCs were CD11c-, and the CD16+ mDCs were all CD11c+. However, CD1c+ mDCs from rhesus macaques expressed low-to-undetectable CD11c (Fig 1E) as we have previously reported [19]. Absolute numbers of DC subsets in uninfected rhesus macaques were 10±6 CD1c+ mDCs/μL blood (mean±SD, n = 21), 61±54 CD16+ mDCs/μL blood and 3±2 CD123+ pDCs/μL blood (Fig 1F). The CD16+ mDCs comprised the majority of mDCs while CD1c+ mDCs were a minor subset.

### CD1c+ and CD16+ mDC numbers are differently modulated with SIV infection, whereas CD123+ pDC numbers persistently decline

**Fig 2:**
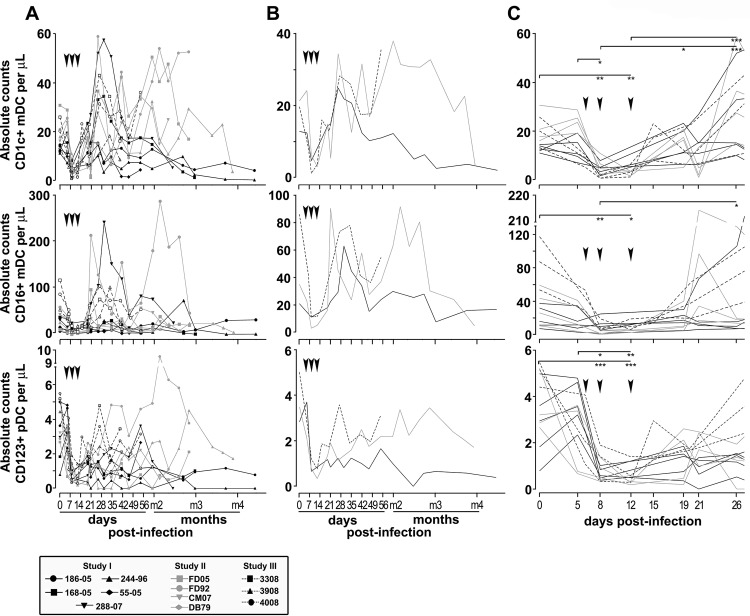
Longitudinal assessment of myeloid and plasmacytoid DCs in whole blood of SIV-infected CD8-lymphocyte depleted rhesus macaques. The absolute cell counts of CD1c+ mDCs (upper row panels), CD16+ mDCs (middle row panels) and CD123+ pDCs (lower row panels) determined over the course of infection are shown. Three independent studies are shown: study I (black symbols and lines; n = 5), study II (grey symbols and lines; n = 4) and study III (black symbols and dotted lines; n = 3). Black arrowheads indicate time of administration of anti-CD8 depleting antibody. **A**. Absolute DC numbers from individual animals throughout infection. **B**. Averages of absolute DC numbers throughout infection. **C**. Individual absolute DC numbers measured in early disease i.e. from pre-infection to 26 dpi including acute (8 dpi) and post-acute phases are shown to emphasize differences between changes in CD1c+ and CD16+ mDC counts. The Kruskal-Wallis test followed by Dunn’s post test was used to determine the significance (asterisks) of differences in absolute numbers during early SIV infection. * *P*<0.05, ** *P*<0.01, *** *P*<0.001. Box shows symbols for individuals animals.

The absolute numbers of CD1c+ and CD16+ mDCs, and CD123+ pDCs were longitudinally analyzed in SIV-infected CD8+ T cell depleted rhesus macaques throughout infection (Fig 2). Absolute numbers of all three subsets of DCs decreased after the first week of infection resulting in a significant loss of CD1c+ mDCs, CD16+ mDCs and CD123+ pDCs (Fig 2A and 2B). However, while CD123+ pDC numbers remained low throughout infection, the numbers of CD1c+ and CD16+ mDCs increased after 3 weeks of infection, with a significant higher increase in numbers of CD1c+ mDCs compared to CD16+ mDCs. A more detailed examination of the first weeks of infection (up to 26 dpi) revealed differential dynamics of absolute numbers of mDCs and pDCs (Fig 2C). Absolute numbers of CD1c+ mDCs significantly decreased by 8 dpi (median: 4 cells/μL [range: 0.6–10.8]; P<0.01) and 12 dpi (median: 3.4 cells/μL [range: 1.2–6.4]; P<0.01) compared to pre-infection (median: 15.2 cells/μL [range: 11–30.6]). Significant increased absolute numbers of CD1c+ mDCs were detected at 19 dpi (median: 13.8 cells/μL [range: 5–23.2]; P<0.05 compared to 8 dpi) and at 26 dpi (median: 29.3 cells/μL[range: 1.2–6.4]; P<0.001 compared to days 8 and 12 p.i.) (Fig 2C). Similar to CD1c+ mDCs, absolute numbers of CD16+ mDCs significantly decreased at 8 dpi (median: 5.7 cells/μL [range: 0.3–24.7]; P<0.01) and 12 dpi (median = 6.1 cells/μL [range: 2.7–18.8]; P<0.05) compared to pre-infection (median: 34.6 cells/μL [range: 7.2–117.4]). Absolute numbers of CD16+ mDCs slightly increased at 26 dpi, one week after CD1c+ mDCs (median: 18.5 cells/μL [range: 7–129]; P<0.05 compared to 8 dpi) but to lower magnitude than CD1c+ mDCs (Fig 2C). This suggests that the replenishment of CD1c+ mDCs occurred at a 2-fold higher rate (within 11 days) than CD16+ mDCs (within 21 days) potentially reflecting a different turnover rate. Absolute numbers of CD123+ pDCs significantly decreased by 8 dpi (median: 0.71 cells/μL [range: 0.3–1.9]; P<0.001) and 12 dpi (median: 0.32 cells/μL [range: 0.2–1.4; P<0.001) compared to pre-infection (median: 3.4 cells/μL [range: 0.8–5.5]) and 5 dpi (median: 3.3 cells/μL [range: 0.7–4.8]) (Fig 2C). In contrast to mDCs, CD123+ pDC numbers remained low at 19 dpi (median: 1.6 cells/μL [range: 0.2–2.6]) and 26 dpi (median: 1.5 cells/μL [range: 0–4.8]). Similar differential dynamics were observed when looking at total percentages of DC subsets (not shown).

**Fig 3:**
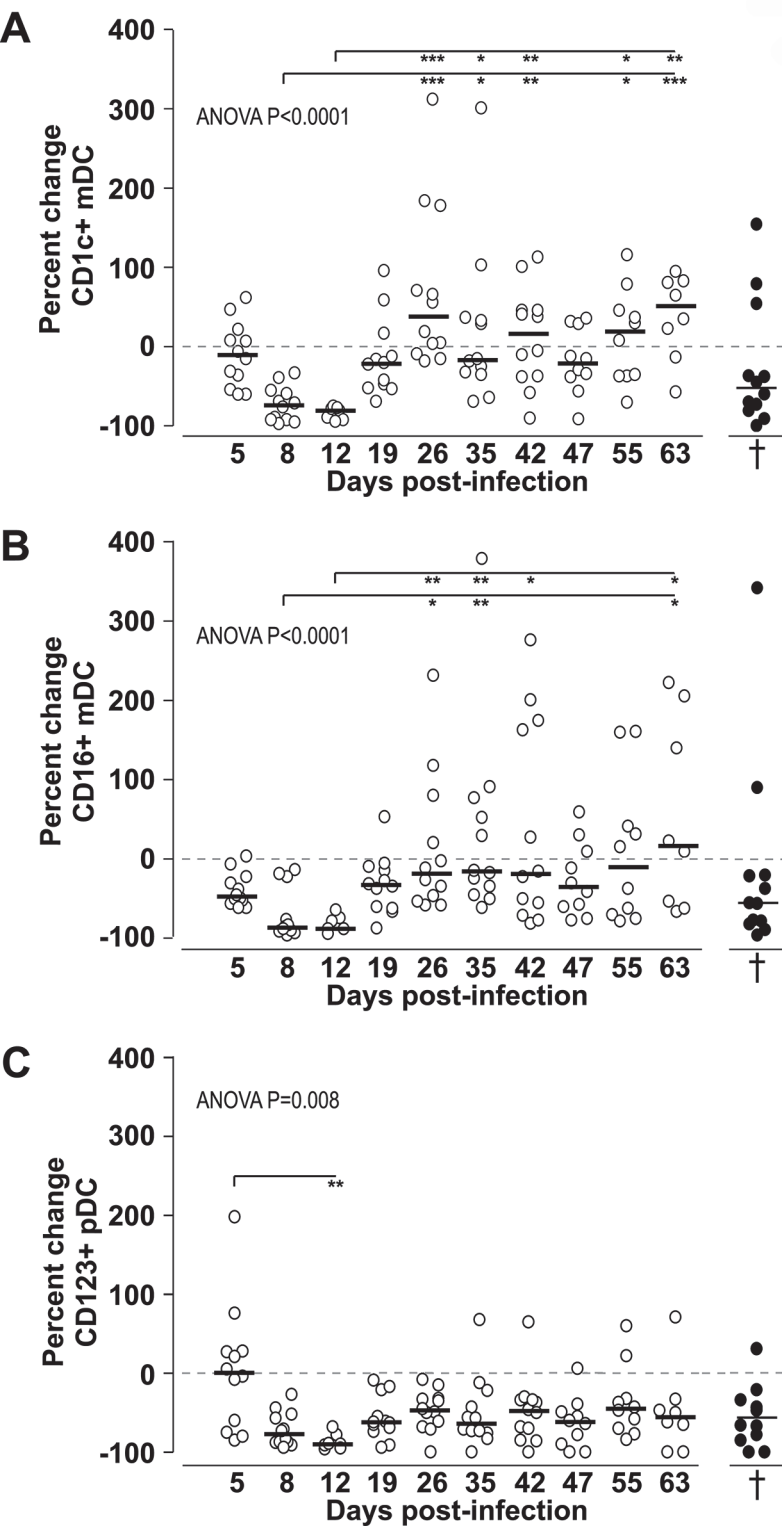
Longitudinal analyssis of the percentage change in absolute numbers of DCs in SIV infected CD8-lymphocyte depleted rhesus macaques. Percent changes in absolute numbers of CD1c+ mDCs (**A**), CD16+ mDCs (**B**) and CD123+ pDCs (**C**) were calculated at different time points post-infection by comparison to pre-infection (baseline: horizontal dashed line “0”). Negative percentages indicate cell loss while positive percentages represent a gain in circulating DCs. Percent changes measured at necropsy time (†) are shown as black symbols. Symbols represent individual animals and horizontal bars represent medians. The Kruskal-Wallis test followed by Dunn’s post test was used to determine the significance (asterisks) of differences in percent change absolute numbers during SIV infection. * *P*<0.05, ** *P*<0.01, *** *P*<0.001.

To better evaluate differences in numbers of circulating CD1c+, CD16+ mDCs and CD123+ pDCs compared to pre-infection, we examined the percent change in absolute numbers over time (Fig 3). Decreased numbers of CD1c+ and CD16+ mDCs occured early at 5 (median: -10.5% and -48% respectively) and 8 dpi (medians: -73.5% and -87.5% respectively) a trend that was reversed by a significant expansion by 26 dpi, especially the CD1c+ mDC population (median: +37.5%; P<0.001 compared to 8 dpi) (Fig 3A and 3B). At this time point, the gain in CD1c+ mDCs was significantly high compared to CD16+ mDCs (P = 0.009, Wilcoxon paired test). These data show that both mDC subsets were repopulated in blood by 3 weeks post-infection. Numbers of CD1c+ mDCs were amplified (median percent changes above 0) while numbers of CD16+ mDCs stayed close to pre-infection counts (median percent changes close to or below 0) suggesting different modulation of these mDC subsets. These differences between the percent changes in CD16+ mDCs and CD1c+ mDCs were significant at 5 dpi (P<0.02) and 26 dpi (P = 0.009) (Wilcoxon paired test). We did not find any differences in percent changes of pDCs over time by 5 dpi. We did not observe any significant rebound of CD123+ pDCs during SIV infection confirming their persistent depletion (Fig 3C).

**Fig 4:**
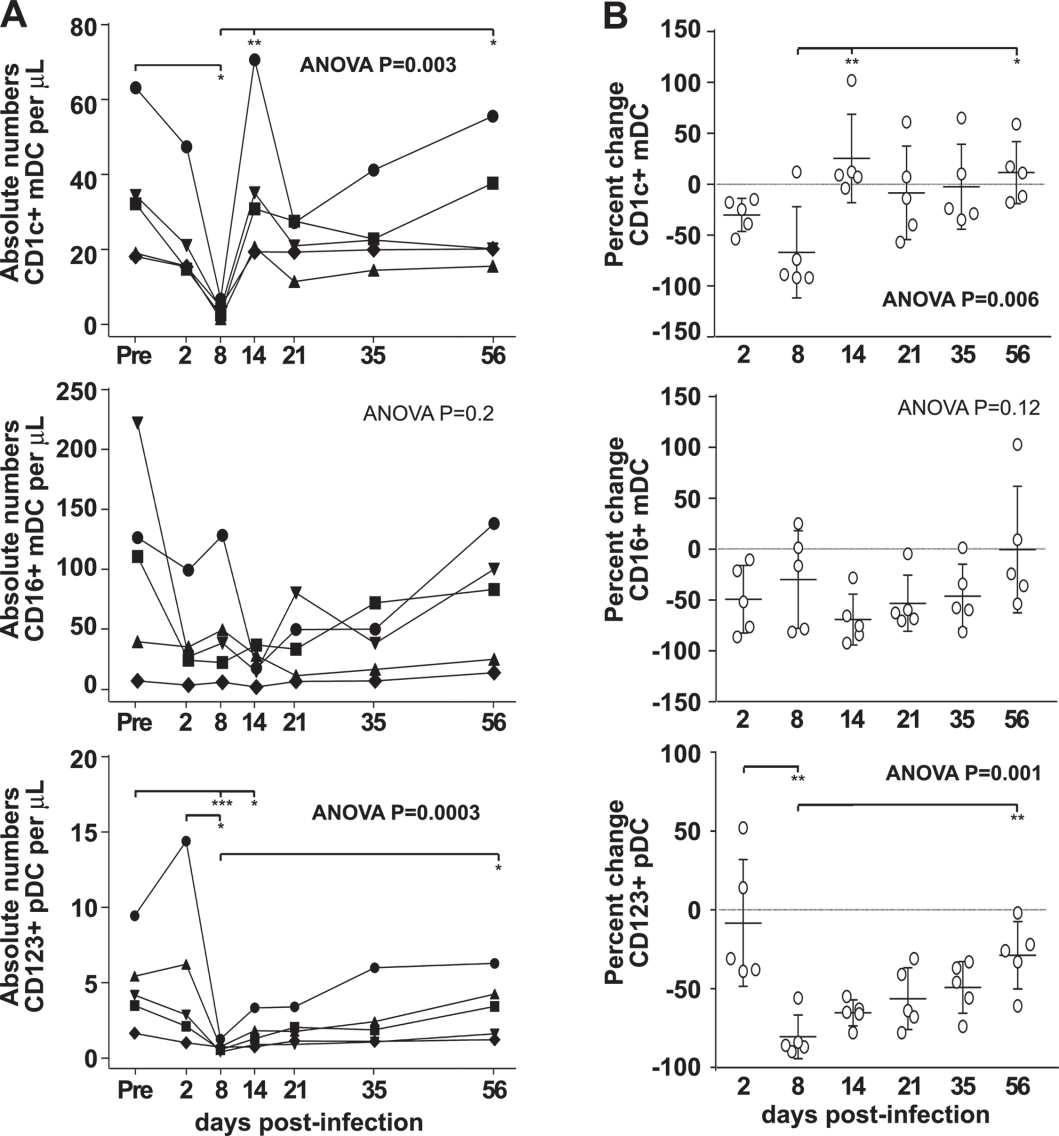
Modulation of absolute counts and percent changes of DCs in SIV-infected rhesus macaques with no CD8 depletion. **A.** Absolute numbers of CD1c+ mDCs CD16+ mDCs and CD123+ pDCs were measured before infection (Pre) and after SIV infection of rhesus macaques without depletion of CD8+ cells. Each symbol represents an individual animal. **B.** Percent changes in absolute numbers of CD1c+ mDCs, CD16+ mDCs and CD123+ pDCs were calculated at different time points post-infection by comparison to pre-infection (baseline: horizontal dashed line “0”). Negative percentages indicate cell loss while positive percentages represent a gain in circulating DCs. Symbols represent individual animals and horizontal bars represent medians. The Friedman test followed by Dunn’s multiple comparison post test was used to determine significant differences in absolute number and percent change during SIV infection. * *P*<0.05, ** *P*<0.01, *** *P*<0.001.

Significant decreases of CD1c+ and CD16+ mDCs and CD123+ pDCs within the first week after infection in the SIV-infected CD8-depleted animals described above correlated with decreases of CD8+ T cells (S1 Fig [26]). In order to exclude the possibility that CD8 depletion alone might be responsible for the changes in DC subsets, we analyzed absolute numbers and percent changes in CD1c+ and CD16+ mDCs and CD123+ pDCs in five rhesus macaques infected with SIV but not CD8+ cell depleted (Fig 4). We observed similar trends in the absolute numbers and percent changes of DC subsets in both models. The absolute number of all three DC subsets were decreased in SIV-infected non CD8 depleted animals within the first week of SIV infection (Fig 4A compared with Fig 2C). Similar to CD8+ T cell depleted animals, the percent change in CD1c+ mDCs in SIV infected animals without CD8 depletion increased at 2 weeks post-infection (median at 14 dpi: +9%) and remained constant (median at 56dpi: +11%). We observed an early loss of CD16+ mDCs (median at 2 dpi: -53%) and CD123+ pDCs at 8 dpi (median: -86%) with both remaining below baseline throughout infection (Fig 4B compared with Fig 3). This suggests that changes in absolute numbers of DC subsets we observed are related to SIV infection rather than compensation for depletion of CD8+ T cells. These data are supported by unpublished observations showing that CD8 depletion alone does not significantly affect the total percentages or absolute numbers of DC subsets over time in uninfected animals (not shown).

### CD16+ mDCs and CD123+ pDCs but not CD1c+ mDCs are significantly decreased with AIDS

**Fig 5:**
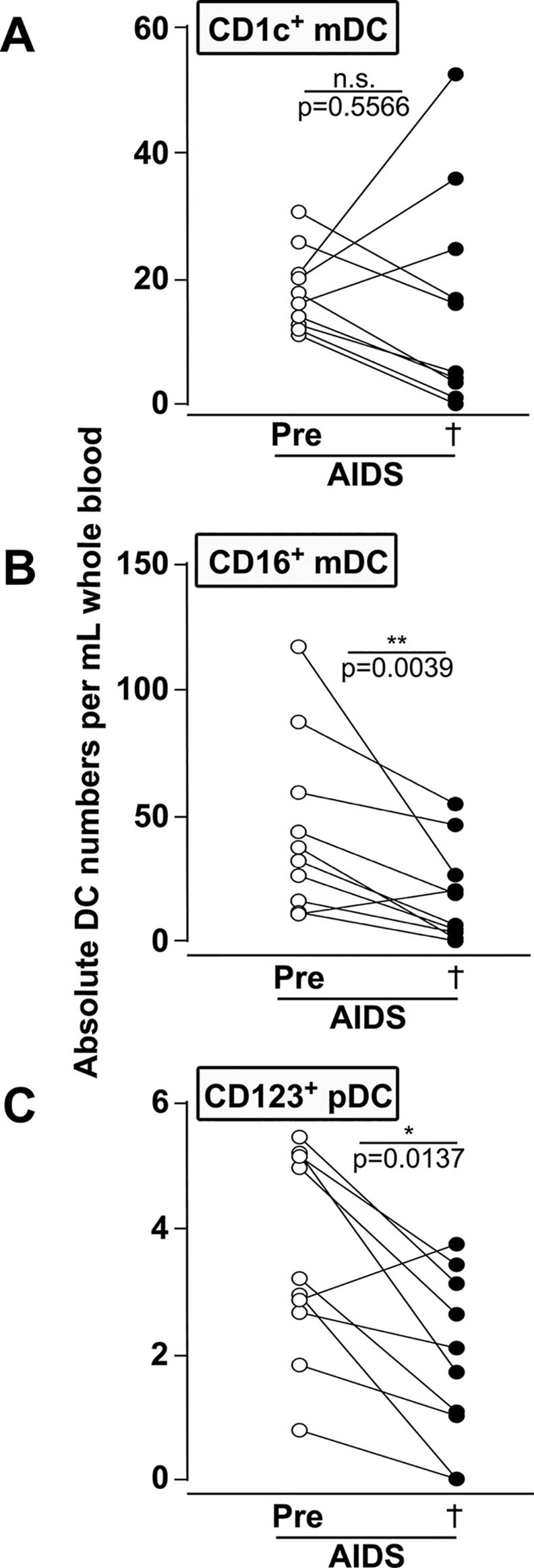
Unlike CD16+ mDCs and CD123+ pDCs, numbers of CD1c+ mDCs with AIDS are not significantly decreased compared to pre-infection. Absolute numbers of CD1c+ mDCs (**A**), CD16+ mDCs (**B**) and CD123+ pDCs (**C**) were measured before infection (Pre, white symbols) and at necropsy (†, black symbols) with AIDS are shown. The Wilcoxon rank sum test was used to perform pair-wise analysis for the absolute numbers of DC before infection and at necropsy with AIDS. P values and asterisks indicative of significant differences are shown; n.s., not significant.

We compared absolute numbers of DCs measured in SIV-infected CD8- lymphocytedepleted animals before infection and at necropsy with AIDS. We did not find any significant differences in the absolute numbers of CD1c+ mDCs with AIDS compared to pre-infection (Fig 5A) probably due to 3 animals out of 10 showing strong increased CD1c+ absolute numbers with terminal AIDS. In contrast, absolute numbers of CD16+ mDCs and CD123+ pDCs were significantly lower with AIDS compared to pre-infection (P = 0.004 and P = 0.01 respectively, Fig 5B and 5C). The same depletion was observed with total percentages of DC subsets (not shown).

### Differential infection of CD1c+, CD16+ and CD123+ DCs *in vivo*

**Fig 6:**
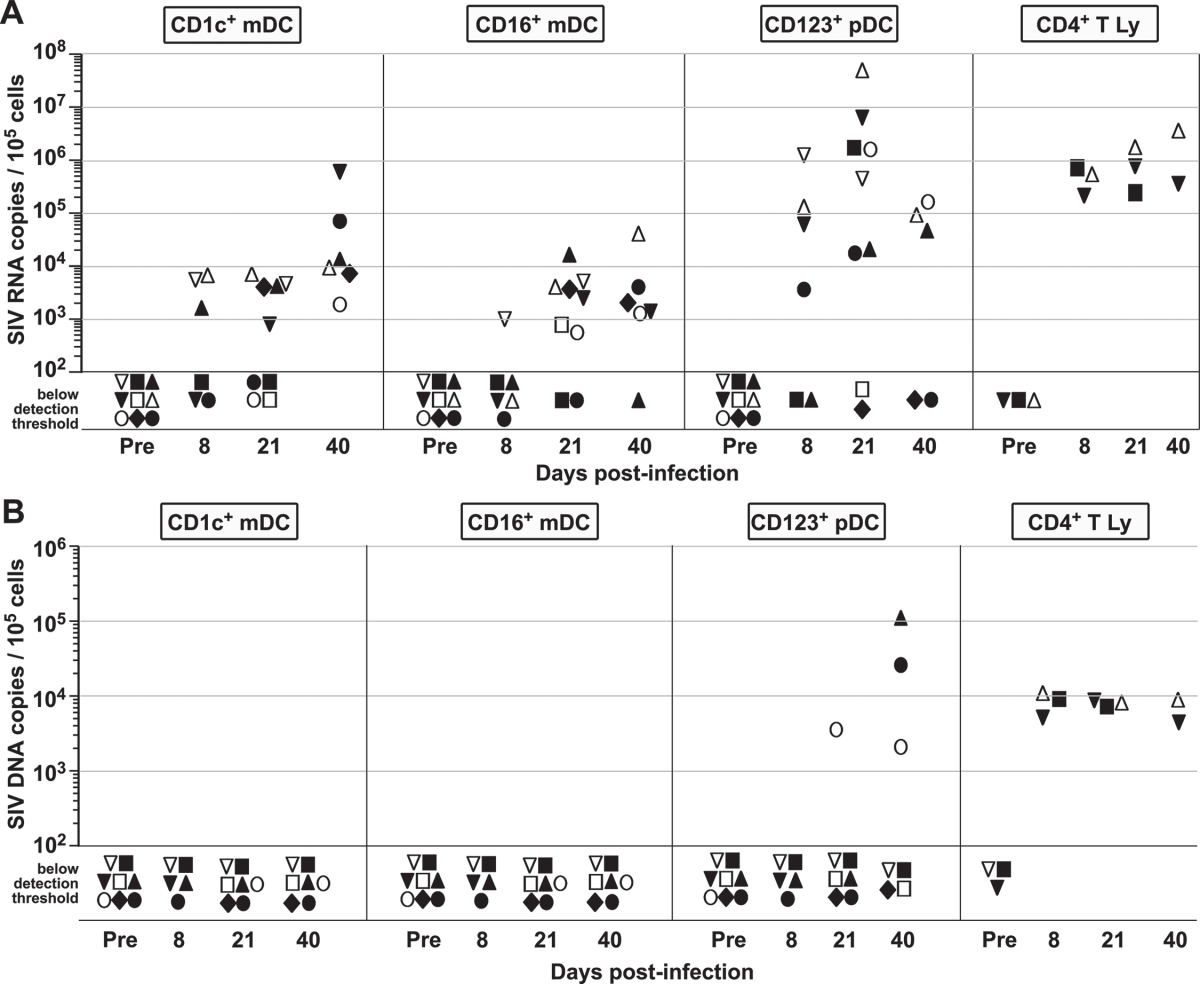
SIV RNA and DNA analysis of FACS-sorted myeloid and plasmacytoid DC subsets from SIV-infected, CD8-lymphocyte depleted rhesus macaques. CD1c+ mDCs, CD16+ mDCs and CD123+ pDCs from were sorted by flow cytometry before infection (Pre) and at days 8, 21 and 40 post-infection (see S2 Fig for gating strategy and post-sort purity analysis). CD4+ T lymphocytes (CD4+ T Ly) from 3 animals were sorted as positive controls for SIV infection. Symbols represent individual animals. Sorted cells were analyzed by PCR for SIV gag. Symbols indicate RNA (A) or DNA (B) copy numbers in samples of sorted DC populations and CD4+ T lymphocytes. Samples that gave negative PCR reaction (limit of the PCR assay: 30 total copies per cell sample) are represented as symbols located below threshold detection. All values have been normalized per 10^5^ diploid genome cell equivalents.

In order to evaluate whether or not blood DCs were associated with SIV (SIV RNA) or active viral replication *in vivo* (SIV DNA), we obtained highly purified CD1c+ mDCs, CD16+ mDCs, and CD123+ pDCs pre-infection and days 8 (acute), 21 (post-acute) and 40 (late stage) after infection. Cells were isolated by FACS using Lin- HLA-DR+ phenotype and non-overlapping expression of CD1c, CD16 or CD123 (S1 Fig). In addition, we isolated CD4+ T lymphocytes as positive controls for cell-associated SIV-RNA and DNA. All sorted samples had cell purity >99%. We detected SIV-RNA in all three DC subsets as early as 8 dpi with variability in RNA levels depending on the DC subset, the stage of disease and the animal studied (Fig 6A). SIV-RNA was detected in CD1c+ mDCs at 8 dpi in 3 samples out of 6 and 21 dpi in 5 samples out of 8 and in all CD1c+ samples analyzed at 40 dpi. SIV RNA was detected in CD16+ mDCs at 8 dpi in only 1 sample out of 6, at 21 dpi in 7 samples out of 8 and at 40 dpi in 5 samples out of 6. SIV-RNA was detected in CD123+ pDCs at 8 dpi in 4 samples out of 5, at 21 dpi in 4 samples out of 5, and at 40 dpi in 3 samples out of 5. CD1c+ and CD16+ mDCs had similar levels of SIV-RNA at 21 dpi and at 40dpi. In contrast, levels of SIV-RNA in CD123+ pDC were significantly higher than in CD1c+ mDCs at 21 dpi (P = 0.02) and in CD16+ mDCs at 21 dpi (P = 0.006) and 40 dpi (P = 0.04). Compared to purified CD4+ T lymphocytes, the CD1c+ and CD16+ mDCs had significantly lower levels of SIV-RNA at 21 dpi (P = 0.04 and P = 0.02 respectively). By contrast, CD123+ pDCs had equivalent SIV-RNA levels compared to CD4+ T lymphocytes (P = 0.4 at 21 dpi). Interestingly proviral DNA was only detected in CD123+ pDCs, late and in only a few samples (1 sample out of 5 at 21 dpi and 3 samples out of 5 at 40 dpi) (Fig 6B). In contrast, SIV-DNA was detected in all CD4+ T lymphocytes (Fig 6B). Levels of SIV-DNA detected in CD123+ pDCs and CD4+ T lymphocytes were similar (Fig 6B). These results suggest that SIV-RNA can be detected in all three subsets of DCs *in vivo* at different levels. mDCs had lower viral loads compared to CD4+ T lymphocytes in contrast with CD123+ pDCs. Only CD123+ pDCs showed SIV-DNA suggesting different susceptibility with SIV infection.

## Discussion

In this study, using SIV infection and CD8-lymphocyte depletion, we examined longitudinal changes in peripheral blood pDCs and mDCs using an original flow cytometry-based approach that allows for the simultaneous detection of non-overlapping CD1c+ and CD16+ mDCs in rhesus macaques. Reports on changes in mDCs during HIV and SIV infection are often contradictory and focused on a unique population of mDCs characterized by CD11c expression, in contrast to the CD11c- CD123+ pDCs [5,10,11]. In addition, the flow cytometry strategies used to identify CD11c+ mDCs are highly variable due to the composition of the antibody lineage cocktail used, gating strategy, limited number of parameters per antibody panel, and use of PBMC vs. whole blood. It is likely that these variations lead to non-DC contaminants and/or overestimation of DC numbers in different studies thus contributing in part to the generation of conflicting data. In addition, there are specific differences within DC subsets. Human CD11c+ mDCs are heterogeneous and include non-overlapping subsets of CD1c+ (BDCA-1), CD16+ and CD141+ (BDCA-3) mDCs [17]. To date, these cells have not been studied as distinct mDC subsets in AIDS with HIV and SIV infection. Moreover, in some studies, CD16 is used as an exclusion marker, which results in the omission of CD16+ mDCs from the analysis. Using a multicolor flow cytometry panel similar to one that we developed for human DC subsets, we detected the following DC subsets in SIV-infected rhesus macaques: CD11c- CD123+ pDCs, and two non-overlapping subsets of mDCs expressing CD1c or CD16 [18,19]. This multicolor analysis strategy gives a more precise phenotypic characterization of mDCs and pDCs by excluding potential contaminants and discriminating CD1c+ vs. CD16+ DCs within the major mDC population. Altogether, we report here that absolute numbers of all three subsets of DCs studied decreased after the first week of infection resulting in a significant loss of CD1c+ mDCs, CD16+ mDCs and CD123+ pDCs. However, while CD123+ pDC numbers remained low in blood throughout infection, the numbers of CD1c+ and CD16+ mDCs increased after 3 weeks of infection, with a significant higher increase in numbers of CD1c+ mDCs compared to CD16+ mDCs. Thus it seems that CD1c+ mDCs and CD16+ mDCs tend to expand in blood three weeks post-infection with a higher mobilization of CD1c+ compared to CD16+ mDCs. In addition, we showed that in contrast to CD1c+ mDCs, CD16+ mDCs are CD11c+ cells. Interestingly, consistently decreasing numbers of CD11c+ mDCs have been reported in SIV-infected animals progressing to AIDS while numbers of CD11c+ mDC remained increasing in stable animals that controlled SIV infection [27]. The CD8 lymphocyte depletion used in this model might have potential effects on numbers of other blood cells including DCs. However, in this study we find similar modulation of the absolute counts of mDCs and pDCs in non CD8 depleted SIV-infected animals suggesting that the CD8 lymphocyte depletion alone is unlikely directly responsible for the changes in DC numbers reported in this study. Transcriptional profiling has shown that CD16+ mDCs are distinct from CD1c+ mDCs even though both cluster with mDCs, in contrast to the CD123+ pDCs, suggesting that CD16+ and CD1c+ mDCs have specialized functions related to their distinct lineage [28]. Accordingly, others reported that CD1c+ and CD16+ mDCs were characterized by different allostimulatory functions and unique responses to Toll-like receptor ligands suggesting that they may potentially have different responses to bacteria or viral infection [17,20]. In humans, CD16+ mDCs have strong proinflammatory activity and produce higher amounts of TNF-alpha whereas CD1c+ mDCs appeared to be mainly inducers of chemotaxis with a strong production of IL-8, a monocyte chemoattractant [20]. Thus, increased numbers of CD16+ mDCs and CD1c+ mDCs observed 3 weeks post-SIV infection might be associated with higher pro-inflammatory and monocyte chemoattractant activities of these mDCs respectively. Increased numbers of both mDCs could then contribute to the expansion and recruitment of circulating monocytes, especially the CD16+ monocytes known to be expanded during HIV and SIV infection [26,29–31]. The changes that we observed in CD16+ mDCs after infection with SIV were similar to those observed by others studying CD11c+ mDCs as a whole population [6]. This can be explained by the fact that CD16+ mDCs represent the main subset within CD11c+ mDCs in rhesus macaques. However, since CD1c+ mDCs in rhesus macaques show low-to-no expression of CD11c in contrast with humans, it is likely that studies reporting changes in CD11c mDCs with SIV infection are excluding the CD1c+ mDC subset from analysis. We also found that absolute counts of CD16+ mDCs were significantly decreased with AIDS compared to pre-infection numbers. Although CD1c+ mDC numbers tend to be lower with AIDS compared to pre-infection, the difference was not statistically significant. Our data might explain in part the conflicting results on changes in mDCs with AIDS since all studies report changes in total CD11c+ mDCs in humans and rhesus macaques without distinguishing between CD1c+ and CD16+ mDCs. The significant decrease of CD16+ mDCs with AIDS is in agreement with reports showing decreases in CD11c+ mDCs [6,11]. Moreover, when we analyzed the absolute numbers of mDCs from the animals in the present study, as a unique population of CD11c+ cells as done in the litterature, we observed a significant decrease with terminal AIDS compared to pre-infection that is almost identical to the one observed with CD16+ mDCs (not shown). Studies reporting no changes or increases in CD11c+ mDC numbers might be biased by the presence of CD1c+ mDCs included in the mDC gate [10,12]. Our results underscore the necessity of distinguishing both subsets within mDC in AIDS studies in humans, and more especially monkeys where CD11c is not a good marker for mDCs, as CD1c+ mDCs in monkeys express low-to-no CD11c [19,32]. The irreversible loss of CD123+ pDCs that we observed early after infection is in agreement with previous reports in humans and monkeys [4,6,10,11,33]. Others reported that this decrease in blood pDCs was probably due to a rapid migration to lymphoid organs [2,4,34].

In this study, we have reported the detection of SIV RNA in CD1c+ mDCs, CD16+ mDCs and CD123+ pDCs as early as day 8 post-infection. Only the CD123+ pDCs had detectable SIV DNA. We found this only during late infection and we only found it in a few samples. Whether blood DCs are productively infected by HIV remains a matter of debate. The paucity of blood DCs and the difficulties to identify/isolate pure populations without contamination by monocytes or T lymphocytes has led to discrepancies about their infectability *in vivo*. Some suggested that blood DCs are not productively infected by HIV-1 [13,14], while others were able to detect HIV-infected mDCs and pDCs, but no integrated virus in pDCs [15,16]. HIV-1 provirus was detected in purified DCs in ART-naive patients but RNA in this study was not analyzed [35,36]. Recent reports suggest that Siglec-1 could be a receptor responsible for HIV capture and storage by DCs and could also play a role in trans-infection of T cells especially after DC maturation [37].

Purified CD123+ pDCs displayed higher levels of SIV RNA than mDCs. It is unlikely that these viral RNA were due to contaminating cells. First, no more than 1% of contaminated cells remained in the sorted samples. Second, we detected CD4+ T lymphocyte-associated SIV RNA ranging 10^5^ to 3.5x10^6^ copies per 10^5^ cells. The levels of SIV RNA detected in CD1c+ and CD16+ mDCs were much lower than those detected in CD4+ T lymphocytes, thus excluding that SIV RNA detected in mDCs are due to lymphocyte contamination. Publications suggesting that circulating DCs are not infected by HIV *in vivo* were focused on determining productive infection. The majority of these studies analyzed proviral DNA as a sign of integration of the virus but omitted viral RNA analysis. SIV RNA detection in mDCs is not unreasonable especially if one considers unspliced genomic RNA. In this case, virions can remain on membrane surface or in non-lysosomal compartment. DCs have the ability to retain virions within surface accessible compartments [38]. The PCR analysis that we used in this study allowed the detection of genomic RNA. Therefore, we report the presence of cell-associated viral RNA on blood DCs without suggesting that these cells are productively infected. Whether SIV RNA is bound to the surface or internalized in vesicles needs to be determined. Finally, we detected SIV DNA only in CD123+ pDCs and in 3 animals out of 7 and late during infection. Levels of SIV DNA detected in CD123+ pDCs were similar to those detected in CD4+ T cells. A recent report showing that pDC in lymph node harbor SIV DNA at a frequency higher than CD4+ T lymphocytes, while SIV DNA in mDCs is undetectable supports, is in a greement with our observations [2]. However, due to the paucity of blood CD123+ pDC especially after 8 dpi, the detection of SIV-DNA in samples of sorted CD123+ pDCs might need further confirmation.

In conclusion, our data demonstrate that CD1c+ and CD16+ mDCs are differently modulated during SIV infection and might play different roles in SIV-associated immunodeficiency. We also show that blood DCs harbor virus *in vivo* but that they seemed not productively infected as suggested by the detection of SIV-RNA only that is probably surface-bound or retained in endosomal compartements, at least on CD1c+ and CD16+ mDCs. Lastly, we would like to underline the importance of considering CD1c+ and CD16+ mDCs as two discrete populations in future studies as they might represent two distinct controllers of the immune response to HIV/SIV infection.

## Material and Methods

### Ethical treatment of animals

This study was carried out in strict accordance with the recommendations in the Guide for the Care and Use of Laboratory Animals of the U.S. Public Health Services/National Institutes of Health, as well as according to the recommendations in the Weatherall report on “The Use of Non-human Primates in Research”. The protocol was approved by the Institutional Animal Care and Use Committee of Harvard University (IACUC ID: 04420; Animal Welfare Assurance Number A3431-01). The rhesus macaques were housed at the New England Primate Research Center (NEPRC, Harvard Medical School, Southborough, MA) in BL2 facilities that are fully accredited by the Association for Assessment and Accreditation of Laboratory Animal Care International. Uninfected animals were socially housed. After infection with SIV, animals were individually housed since animals with more advanced disease may not be able to compete for food. It is also more difficult to evaluate and monitor the health status of the animals in social housing. Compensatory enrichment was provided to animals that were not socially housed. Enrichment was provided through manipulatable devices, foraging opportunities, food items, structural and environmental enhancements and positive human interaction. Enrichment devices were rotated on a weekly basis and include toys, mirors, radios, TV/VCRs, foraging boards, and a variety of complex foraging devices. Cages were positioned so that animals had visual contact with other animals. Animals were observed daily by animal care staff for signs of illness related to SIV infection including inactivity, anorexia, diarrhea, and dyspnea. Veterinary staff were immediately notified if abnormalities were observed. Euthanasia were performed based on the following criteria: weight loss >15% in 2 weeks or 30% body weight in 2 months or 25% overall, documented opportunistic infection, persistent anorexia > 3–5 days without explicable cause, severe intractable diarrhea that is nonresponsive to standard treatment and results in dehydration and debilitation of the animal, progressive neurologic signs—i.e. instability on the perch bar, depression, head tilt, nystagmus, ataxia, stupor, or depression, significant cardiac and/or pulmonary signs—i.e. dyspnea, open-mouthed breathing, severe, previously unrecognized, cardiac murmur especially if resulting in pulmonary edema, progressive or persistent anemia, or body condition score <1.5/5 with weight loss. All animal procedures were performed under ketamine or telazol anesthesia to minimize discomfort and stress.

### Animals and experimental protocol

Three independent studies were conducted with 12 adult rhesus macaques (*Macaca mulatta*): study I (n = 5), study II (n = 4) and study III (n = 3). Animals were infected intravenously with SIVmac251 on day 0. CD8+ lymphocytes were depleted by subcutaneous administration of an anti-CD8 monoclonal antibody (cM-T807; provided by Dr K. Reimann) on day 6 post-infection (p.i.) followed by two intravenous infusions on days 8 and 12 p.i. CD8+ T lymphocyte depletion results in accelerated development of AIDS within 3 months post-infection [21,39]. Following depletion in CD8+ lymphocytes, plasma virus peaked from 10^7^ to 10^8^ copies per ml by 14 days p.i. and remained at or near those levels throughout the course of study in all 12 animals by 7–14 days p.i. (S1A Fig) and remained constant throughout the infection as previously decsribed [21,26]. All but two animals (3308 and 186–05) were persistently CD8+ lymphocyte-depleted (>28 dpi) (S1B Fig). AIDS rapidly developed in the 10 out of 12 animals with persistent depletion within 3 months post-infection (median = 76 days [42 to 131 days]; n = 12). The diagnosis of AIDS was determined by the presence of AIDS defining lesions: Pneumocystis pneumonia, Mycobacterium avium infection (most commonly small intestine, liver and mesenteric lymph node), and intestinal adenovirus infection (most common in small intestine). Other, less common lesions include SIV giant cell disease in the lung, gut, and lymph nodes and SIV associated arteriopathy. Whole blood was collected in ethylenediaminetetraacetic acid (EDTA) before SIV infection (pre) and at different time points after SIV infection until necropsy. Using Wilk’s lambda multivariate analysis of variance (MANOVA), we determined no significant differences between absolute cell counts and percent changes of CD1c+, CD16+ and CD123+ DC subsets in studies I, II and III (*P*>0.05). For these reasons, data from these three studies were pooled. In addition, five rhesus macaques that were infected with SIVmac251 but not CD8 depleted were used to control possible effects of CD8 depletion on absolute numbers of mDCs and pDCs.

### Flow cytometry: phenotype analysis and cell sorting for detection of SIV RNA and DNA

#### Antibodies

A cocktail composed of the following monoclonal antibodies was used: anti-CD16-FITC (clone 3G8), anti-CD141-PE (clone 1A4), anti-CD123-PerCP-Cy5.5 (clone 7G3), anti-CD3-PE-Cy7 (clone SP34-2), anti-CD14-Pacific Blue (clone M5E2), CD20-APC-Cy7 (clone L27) all from BD Pharmingen (San Jose, CA), anti-CD1c-APC (clone AD5-8E7, Miltenyi Biotec, Auburn, CA), anti-HLA-DR-PE-Texas Red (clone Immu-357, Beckman Coulter, Miami, FL), anti-CD11c-Alexa700 (clone 3.9, eBiosciences, San Diego, CA), anti-CD8-Qdot 655 (clone 3B5, Invitrogen, Carlsbad, CA) and anti-CD4-Qdot 605 (clone S3.5, provided by Dr K. Reimann).

#### Eleven-color flow cytometry

Erythrocytes in 100μL of whole blood were lysed using Immunoprep reagent on a T-Q prep machine (Beckman-Coulter, Fullerton, CA). We routinely use two 100μl samples of whole blood in separate tubes to ensure obtain optimal numbers of DC. After lysis, leukocytes from two tubes were pooled, washed with phosphate buffered saline (PBS) containing 2% fetal bovine serum (FBS) and incubated with a pre-mixed antibody cocktail described above for 15 minutes at room temperature in the dark. Stained cells were washed with PBS-2% FBS, and resuspended with freshly prepared 1% paraformaldehyde (PFA) and analyzed on a BD FACS Ariaflow cytometer (BD Biosciences) as previously described [18]. One million total events were collected for analysis. Absolute cell numbers of each subset in blood were calculated by multiplying the total percentage of cells by the number of white blood cells per microliter of blood as determined by complete blood cell counts. Data were analyzed using FlowJo software (version 7; Treestar, Ashland, OR).

#### Cell sorting

CD1c+, CD16+ and CD123+ DC subsets were sorted from peripheral blood mononuclear cells (PBMCs) by flow cytometry. Briefly, PMBCs were obtained by density gradient centrifugation (Ficoll-Paque PREMIUM; GE Healthcare Biosciences, Piscataway, NJ) and were incubated with a mix of the following antibodies: anti-CD11c-PE, anti-HLA-DR-PE-TexasRed, anti-CD123-PerCP-Cy5.5, anti-CD16-PE-Cy7, anti-CD1c-APC, anti-CD3-APC-Cy7, anti-CD20-APC-Cy7, anti-CD14-APC-Cy7 and anti-CD8-Qdot655. DC sorting was performed on a FACSAria equiped with 3 lasers (Becton Dickinson) modified as previously reported [19]. We sorted between 190–70,000 CD1c+ mDCs (median 3,200 cells), 3,300–110,000 CD16+ mDCs (median 19,000 cells), and 160–4,700 CD123+ pDCs (median 1,900 cells) at the following time points: 1) before infection, 2) day 8 (acute), 3) day 21 (post-acute) and 4) day 40 (late stage) p.i. Because the number of cells, especially the CD123+ pDCs sorted from the infected animals was too low for a post-sort analysis, we performed in parallel the same sort on an uninfected age-matched animal using the same cell sorting parameters to assess the purity of sorted populations. Sorted cell populations from the uninfected animals were analyzed after sorting and the purity of all sorted populations was >99% with less than 0.1% of CD4+ T cell contamination.

### Viral loads

Plasma and cell-associated viral loads were determined as previously described [40,41] by quantitative PCR methods targeting a conserved sequence in gag. The threshold detection limit for 0.5 mL of plasma typically processed is 30 copy equivalents per mL. The threshold detection limits for cell associated DNA and RNA viral loads are 30 total copies per sample, respectively, and are reported per 10^5^ diploid genome cell equivalents by normalization to a co-determined single haploid gene sequence of CCR5.

### Statistical analysis

Kruskal-Wallis non-parametric test followed by Dunn’s post-test was used for multiple comparisons of percent changes between time points. Non-parametric Wilcoxon matched pair test was used for comparisons of absolute cell numbers between pre-infection and necropsy times. Differences in cell counts were considered statistically significant with P values <0.05. Correlations were determined using Spearman non-parametric test, where two-tailed p values <0.0001 were considered significant at an alpha level of 0.05. Statistical analyses were computed with Prism software (version 5.02; GraphPad Software, La Jolla, CA). Multivariate analysis of variance (MANOVA) and general linear model of regression were computed with SAS/STAT software (SAS Institute Inc., Cary, NC).

## Supporting Information

S1 Fig Long-term depletion of CD8+ lymphocytes in SIV-infected rhesus macaques induces persistent increased plasma virus. **(A)** Virus (SIV-RNA gag) was quantified in plasma samples by RT-PCR at different time points. Each line indicates an individual animal. Three independent studies are shown: study I (black symbols and lines; n = 5), study II (grey symbols and lines; n = 4) and study III (black symbols and dotted lines; n = 3). **(B)** Longitudinal analysis of absolute numbers of CD3+CD8+ lymphocytes from SIV-infected CD8+ lymphocyte-depleted rhesus macaques from pre-infection (day 0) to necropsy time. Two animals (186–05 and 3308) were transiently CD8+ lymphocyte depleted (<28 days) and 10 animals were persistently CD8+ lymphocyte depleted (>28 days). Box shows symbols for individuals animals.
(TIF)

S2 Fig Gating strategy for DC sorting and purity analysis. **(A)** Gating strategy. DCs were selected according to FSC/SSC properties. Lin- cells such as CD14+, CD20+ and CD3+ cells were excluded and HLA-DR+ were selected. From this Lin- HLA-DR+ population, CD1c+ mDCs, CD16+ mDCs and CD123+ pDCs were sorted. From the CD3+CD14-CD20- cell population, CD4+ T lymphocytes were sorted as positive control cells for cell-associated SIV. **(B)** Post-sort analysis of the purity of sorted cells.
(TIF)
