# Supplementary figures and images for: Distinct Phenotype, Longitudinal Changes of Numbers and Cell-Associated Virus in Blood Dendritic Cells in SIV-Infected CD8-Lymphocyte Depleted Macaques
Source: PLoS One. 2015 Apr 27;10(4):e0119764. doi: 10.1371/journal.pone.0119764 (PMC4410956; doi:10.1371/journal.pone.0119764)

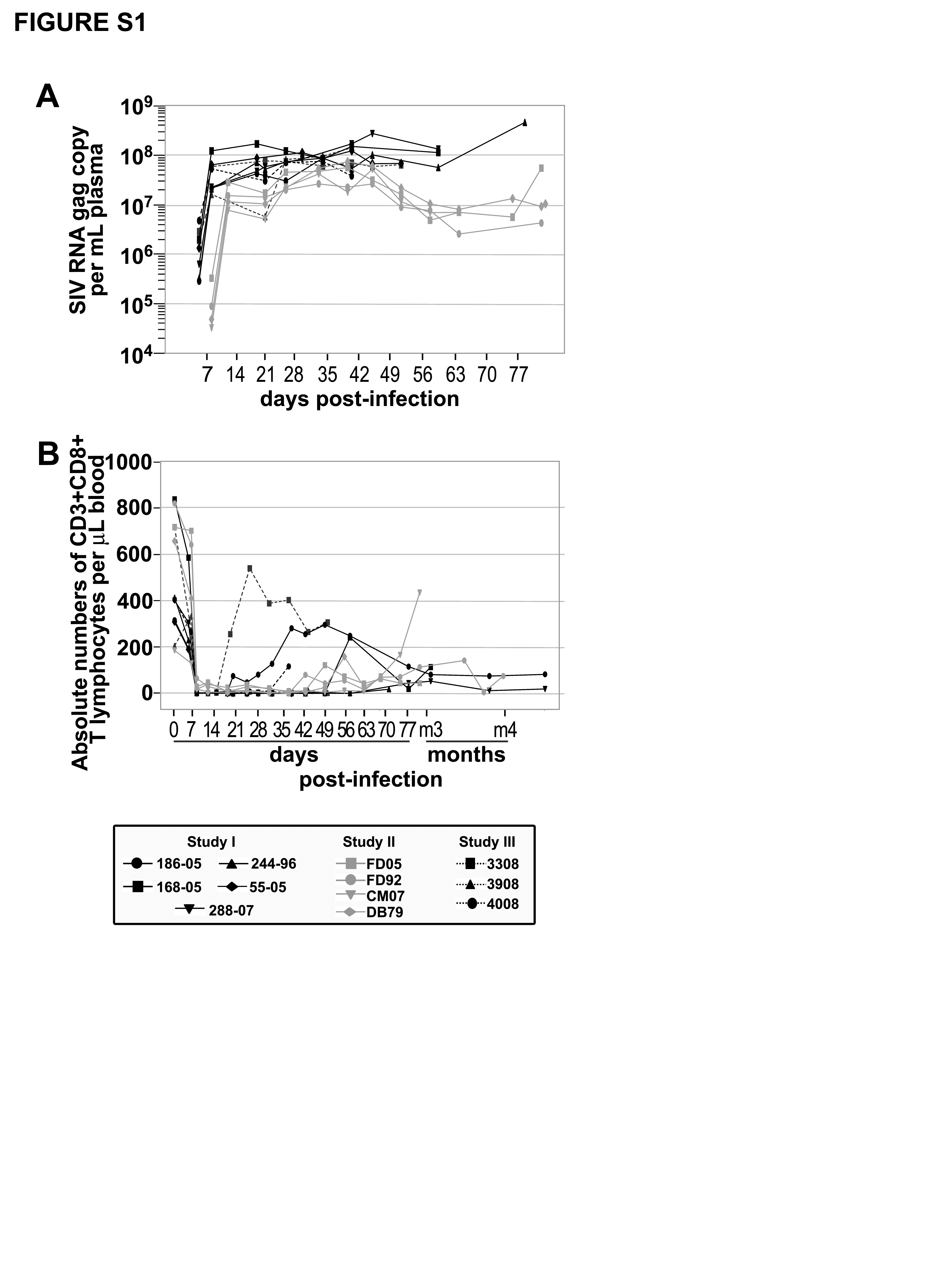

Supplement: S1 Fig — (A) Virus (SIV-RNA gag) was quantified in plasma samples by RT-PCR at different time points. Each line indicates an individual animal. Three independent studies are shown: study I (black symbols and lines; n = 5), study II (grey symbols and lines; n = 4) and study III (black symbols and dotted lines; n = 3). (B) Longitudinal analysis of absolute numbers of CD3+CD8+ lymphocytes from SIV-infected CD8+ lymphocyte-depleted rhesus macaques from pre-infection (day 0) to necropsy time. Two animals (186–05 and 3308) were transiently CD8+ lymphocyte depleted (<28 days) and 10 animals were persistently CD8+ lymphocyte depleted (>28 days). Box shows symbols for individuals animals. (TIF) [file pone.0119764.s001.tif]

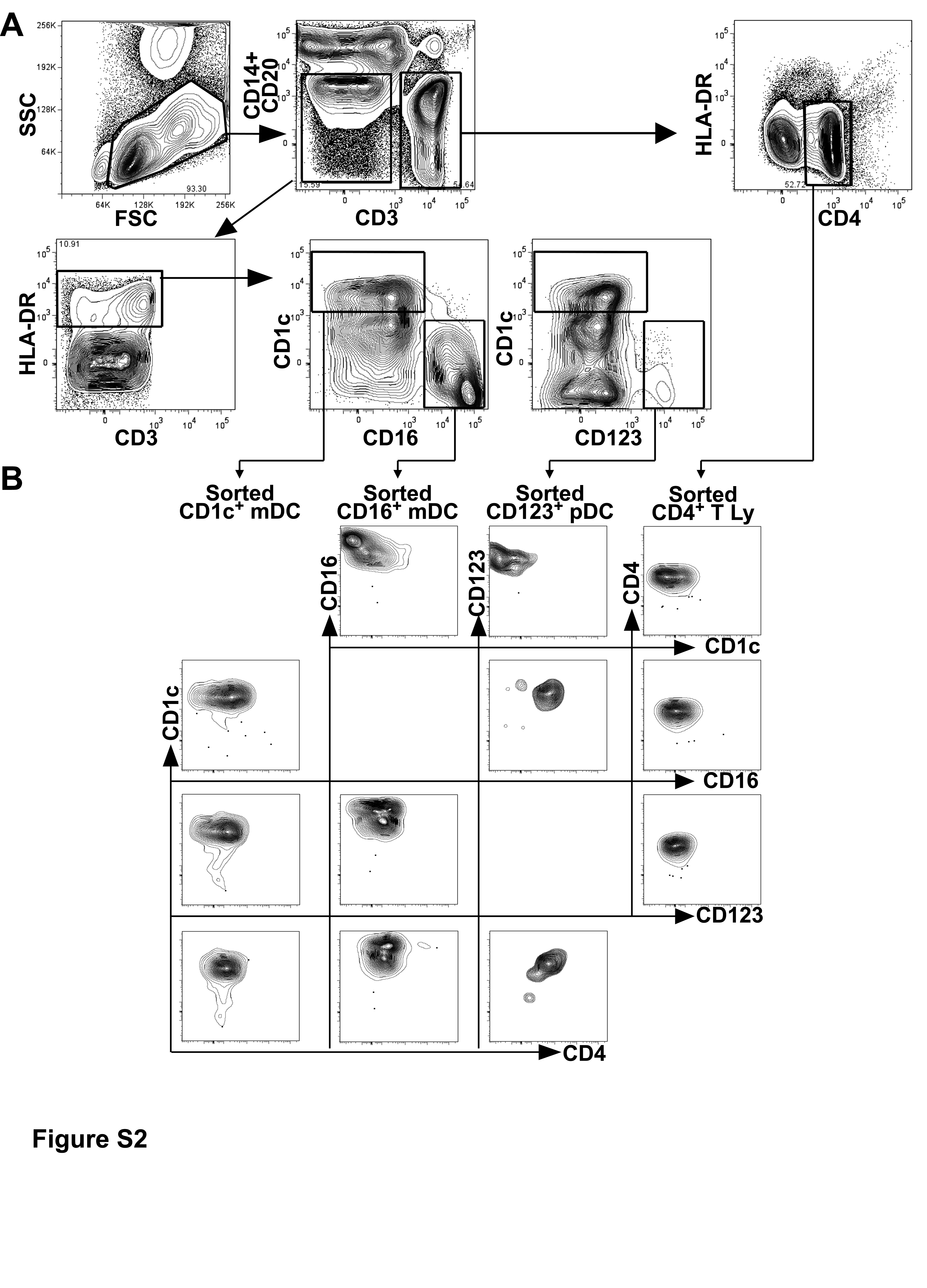

Supplement: S2 Fig — (A) Gating strategy. DCs were selected according to FSC/SSC properties. Lin- cells such as CD14+, CD20+ and CD3+ cells were excluded and HLA-DR+ were selected. From this Lin- HLA-DR+ population, CD1c+ mDCs, CD16+ mDCs and CD123+ pDCs were sorted. From the CD3+CD14-CD20- cell population, CD4+ T lymphocytes were sorted as positive control cells for cell-associated SIV. (B) Post-sort analysis of the purity of sorted cells. (TIF) [file pone.0119764.s002.tif]
